# Kindness leadership needed now more than ever, in global health and beyond?

**DOI:** 10.7189/jogh.14.03013

**Published:** 2024-05-31

**Authors:** Janet Michel, Odile Pham-Tan, Marcel Tanner

**Affiliations:** 1 Swiss Tropical and Public Health Institute (Swiss TPH), Basel, Switzerland; 2 University of Basel, Basel, Switzerland

If the pandemic taught us anything, it is how contested and important leadership is. Researchers have challenged the sacredness of leadership and offered alternatives to bridge the separation between leaders and followers. According to distributed leadership, leadership actors can emerge from anywhere in an organisation [1]. A leader is defined as a person who has a vision, a path to realising it, someone who inspires, motivates and supports followers to achieve and reach those goals [2]. The role of a leader changes according to the size and composition of a team among other factors [3].

The issue of persona in leadership is regaining attention. Can a leader have one persona at home and another at work? This phenomena has been proven otherwise [4]. A person brings the whole self to work, idiosyncracies and all. Personality influences the way individuals think, act and feel in any given situation, be it at home or at the workplace [4].

The five big personality traits believed to be the building blocks are:

• Will, agreeableness – the driving force behind the promotion and defence of one’s own ideas.

• Control, conscientiousness – the amount of self-discipline and responsibility.

• Energy extraversion – the extent to which one needs to interact with other people.

• Affection – openness to experience and the degree to which one is focused on self or others.

• Emotionality – neuroticism-the level of tension and apprehension in everyday life.

Among these, energy has been found as a strong predictor of leadership. People who score high on energy are enthusiastic, energetic, sociable, imbued with the ability to command a room, motivate, and inspire others.

The coronavirus disease 2019 (COVID-19) pandemic crisis was also a leadership crisis, according to Tourish 2020 [5]. He argues that decision making with poor evidence is hazardous [5] and is a sign of leadership failure. Others state that mainstream leadership and theories are not useful in an environment of radical uncertainty [5]. COVID-19 was certainly an environment of uncertainty. The argument is that populist leaders exploit uncertainty by suggesting simple solutions [5] without any accountability.

Regardless of who is right or wrong, we think that the pandemic is an opportunity to explore how the theory and practice of leadership can contribute to better outcomes [5]. The responses of formal leadership in response to COVID-19 were viewed by some as focussing on bringing under control, a virus that had gone out of control, justifying that a state of perfection can be achieved through calculated and rationalised leadership control [1]. The challenge of this approach is the oversimplification and the regard of all problems as puzzles rather than mysteries [1]. We would like to view COVID-19 as a mystery, certainly in part. Social distancing and other measures were put in place in an attempt to protect some segments of the population, like the elderly and those with co-morbidities. Societal divisions, alienation and mental health issues have been documented as some of the unintended effects of these measures [6,7]. The situation has been unprecedented and learning from what happened can prepare us better as pandemics are forecasted to increase.

## IN THE SEARCH FOR THE HOLY GRAIL

In contrast to an earlier statement, Marsh 2021 [3] argues that identifying leaders is not only about high energy or high control people, as anyone can be an exceptional leader. Leadership development however, has the responsibility to help employees gain self-awareness and to coach them to excel at this [3]. The holy grail seems to be emotional intelligence accompanied by affection [8]. Emotional intelligence is defined as the ability to read the room and realise the best way to motivate the team. Affection is associated with leadership because people with high affection are interested in other people’s feelings and interests and are more likely to know how to inspire and motivate them [3]. Emotional intelligence has also been found as key in delivering constructive feedback, in collaborating with others, coaching and stress management [9]. Equally important is that employees seem to feel safe and comfortable where leadership is kind and sensitive, making them effective and creative. This underscores the importance of diversity and inclusion at the work place, inviting individuals to be themselves at work [10]. Kindness leadership seems to summarise the above qualities. It is defined as intentional leadership with a clear understanding of how leaders conduct themselves and treat staff. Kind does not mean weak, and bold and assertive does not mean a good leader.

## GENDER ISSUES IN LEADERSHIP

Men, in general, are more likely to be promoted to leadership positions than women, bearing in mind that only 16% of CEO positions are occupied by women [11]. These obstacles to success seem to increase the risk of women adopting masculine work persona-subscribing to the ‘alpha male’ culture that typically dominates work places. The female alpha male attitude seems to grant women that get into leadership acceptance, protection, promotion and clout [11]. The clash seems to be between communal and agentic qualities. Women tend to exhibit communal qualities of concern, compassion, affection, helpful, friendly, kind, sympathetic, interpersonal sensitivity and gentleness [11]. Men are associated with agentic behaviours of assertion, control, aggressive, competitive, dominance, self-confident, forceful, self-reliant and individualistic [11]. Looking at the above, which behaviours are associated with a toxic workplace? Any wonder work places are making people sick [12–14]?

## EMOTIONAL INTELLIGENCE

According to literature, introverts and extroverts are equally effective as leaders [4]. Again, what seems critical though is emotional intelligence – the ability to read the room and employ the best approaches to motivate the team [4]. The reasons introverts are viewed as good leaders is their tendency to be good listeners, embrace solitude, meaning having time to think things through, research and write, making them great at preparing ahead. They also tend to be calm and exhibit a sense of self-confidence [4]. Regardless of being introverts or extroverts, exceptional leaders have been found to have one quality in common, self-awareness [3]. A leader with self-awareness knows what trips subordinates up, takes action and adapts behaviour [3]. A leader with self-awareness knows their strengths, granting them confidence and their weaknesses, making them get the right people around them [3].

## BURN OUT AND MASSIVE RESIGNATIONS

In recent years, there seems to be a lot of focus on strength and power at the workplace, making leaders lose sight over the need for kindness [1]. Sixty percent of health care workers come to work when unwell [15]. More than half of global physicians and nurse work when they have flu-like symptoms, a real concern now in the era of COVID-19 [15]. The soldier-on mentality, where one feels indispensable, fear of letting the team down or doing a disservice to the community by taking time off, might be to blame [15]. The pressure from supervisors, calling for health care workers to report for work even when sick, cannot be ignored either [15]. A link between sickness presenteeism-working while sick and mental health has been found amongst health care workers including doctors. This is compounded by the fact that mental health is an unseen illness with substantial stigma and shame among the medical community [15]. The information overload has also taken humanity and instincts out of the work place, making it artificial and over engineered [16], creating additional stress. The current burn out and work-related stress particularly in the health sectors, calls for a shift in our thinking and our way of doing things.

## KINDNESS TAKEN OUT OF THE WORKPLACE

Kindness at the workplace has been viewed as a weakness, dampening decisive decision making [17]. According to research, leaders can be both empathic and strong [16]. Kindness means good business [17,18]. Kindness has been identified as the hall mark of people led leadership and kindness is strength [17]. Kindness also means intentional leadership with a clear understanding of how leaders conduct themselves and treat staff. People with soft skills are capable of making good and tough decisions with kindness. In support of our assertions, kindness has been found to increases happiness, success and innovation at the workplace [17–21]. Kindness leadership embodies authenticity, transparency, warmth, trust building and empowers people [17]. Kindness leadership is demonstrated by kindness, caring, gentleness, and graciousness towards colleagues [21]. Kindness leadership competencies include team work, motivation, risk taking and mindfulness [21]. Going by the attributes of kindness leadership described above, workplaces today seem deficient of compassion and kindness.

## EVIDENCE FOR KINDNESS LEADERSHIP AND FEMININE ATTRIBUTES

Research has established a key link between stress management and emotional intelligence [9,22]. To capitalise on this, a behavioural and cultural shift is needed [15] calling for a new type of leadership. Kindness leadership has been shown to improve well-being at the work place. Well-being at the work place is defined as self-perceived health well-being including the absence of negative experiences and stressors [23,24]. Leadership affects employee well-being, regardless of off-site or on site work [25]. Further studies also revealed that kindness leadership affects trust between subordinates and managers as well as employees’ sense of meaning in the work [26]. One study of 204 employees found evidence associating kindness leadership with a positive relationship to employee job performance, mediated by supervisor-subordinate guanxi (SSG). Supervisor-subordinate guanxi is described as the relationship between a supervisor and a subordinate. In the study, SSG was positively associated with job performance (r = 0.50, *P* < 0.01) [27].

## WHAT KINDNESS LEADERSHIP LOOKS LIKE IN PRACTICE

Kindness leaders do the following:

• Encourage participation in work teams.

• Appreciate their subordinates, care about them and have full confidence in what they do and allow their team members to give their opinion.

• Have a positive attitude towards solving problems and complex situations.

• Feel comfortable with their work and do it for personal satisfaction.

• Keep their subordinates motivated, which makes them different.

• Enhance the work environment by encouraging initiative and innovation.

• Spend time with each team member to find out how they are doing and to remind them of the common goals they have set.

• Embrace complex situations as they tend to identify a problem, reflect, consider possible solutions and act.

• Are not afraid to fail, as they see a mistake as a learning experience [21].

Kindness leadership is the type of leadership needed today more than ever. Though the alpha male persona seems to be the expectation from women aspiring to be leaders, many have discovered that morphing of personality can damage personal authenticity and turn one`s purpose from something noble to a ruthless ambition devoid of meaning [11]. This raises the question: ‘Is it worth it?’ It is increasingly evident that people perform better when they are themselves, authentic, accepted, and valued. Authenticity enhances wisdom and self-belief [10,11]. A 2015 study found that 34% of employees thought that women make honest leaders and better mentors. Openness and empathy were qualities that were associated with why women got better results in leadership [11,22,28]. Would women and men alike not do better by moving away from competitive, cut throat tactics to creating work environments where empathy, honesty, ability to listen are valued as assets and not weaknesses [11]? Is the pandemic not an opportunity to do so? If not now when then?

## MENTAL HEALTH AND ‘BURN OUT’

In our view, the mental health issues in the society, massive resignations and ‘burn outs’ all point at compassionate fatigue workplaces. Our work places seem to be making people sick. Competition, back biting and elbowing are rampant. While work has become physically less dangerous, it has become more psychologically damaging [12]. In the UK, the number of disability claims have doubled in a year [12]. Unrelenting hours, capricious bosses, lack of control, toxic colleagues, unrealistic deadlines [13] have been identified as characteristics of toxic work places. Are bosses responsible? Research revealed that our brain and body have trouble distinguishing between stress caused by a real danger like a house on fire and stress caused by a boss with too many demands [14].

The current stress-ridden work place seems to be calling for kindness in leadership, not being afraid to be kind and allowing oneself to be driven by empathy. Assertiveness and strength have been viewed as incompatible with kindness and empathy [16]. Empathy is a skill that can be taught [29]. Focusing on being assertive and strong, makes leaders lose oversight of why they are in the leadership position in the first place, for others and not themselves [16]. Learning to filter things and acknowledging that the leader and the other person may have different perspectives is the key. Sensitivity, gratitude and forgiveness for oneself and others have also been identified as paramount [16].

*The world does not need a whole lot of massively thick-skinned politicians; it needs a world with people who care. –* Jacinda Arden (Prime Minister of New Zealand)

**Figure 1:**
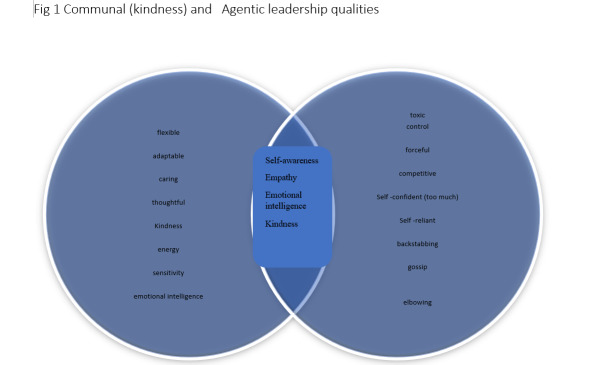
Communal (kindness) and Agentic leadership qualities.

Kindness leadership is indeed good news and encouraging for everyone, particularly women in leadership. Women in leadership positions tend to adopt ‘alpha attitudes’ to fit in, thrive or be viewed as serious and capable. Competition rather than cooperation has been revealed, with women in leadership perpetuating toxic work place behaviours, gossip, elbowing and backstabbing [4,12,13] to be accepted and viewed as serious. There is place for communal, feminine leadership at the work place. The CEO of General Motors Mary Barra, is considered one of the most powerful women in the world and has successfully driven the company by her quiet, humble and kindness leadership the past five years [17]. Jacinda Arden the prime minister of New Zealand exudes warmth, kindness and compassion becoming an iconic and respected leader of our time [17] (**Figure 1**). Another discussion beyond the scope of this viewpoint, is which of these attributes can be taught or learnt and which attributes are inborn.

If toxic behaviour at the work place is left unchecked, it will spread and kill the organisation. The only antidote is strong, positive and kindness leadership [30].

*Kindness leadership is about being in charge but not controlling. It is not about creating great things, but about doing the ordinary things with the conviction, appreciation and joy of and for their basic intrinsic values. –* Prof Marcel Tanner, farewell lecture at the University of Basel [31]

## OPPORTUNITIES AND CHALLENGES

Kindness leadership in the era of digital transformation, pandemics, social, economic, climate environmental and political uncertainty among them wars, is a glimmer of hope in the sea of ensuing hopelessness [21]. Needless to say, not all managers are comfortable with the idea and some fear that kindness leadership could have unintended consequences [32]. This is not unusual as change is always associated with fear and uncertainty. Research is needed to explicitly examines if and how kindness leadership can be taught and how the outcomes can objectively be measured. Some components associated with kindness leadership, for example mindfulness, have been a subject to criticism of maybe being of benefit to some while upsetting the others [32,33]. Additional research is required to verify these claims. It is important however to highlight that kindness leadership is not synonymous with mindfulness.

## CONCLUSIONS

Kindness leadership is quickly being seen as essential for success, competitiveness and innovation [17,18,21]. We bet it is time. If the pandemic has taught us anything, it is that staff are valuable assets, people that also need to be taken care of. We also have learnt that kindness leadership can keep staff motivated, clients satisfied and the organisation performing highly. The glaring and irrefutable evidence is that our work places are toxic, making people sick [12,13,34]. Mental health issues have shot through the roof. Where is the leadership? Time to invest in the creation of a work place culture of kindness? From the above discussion, leadership emerges as both a verb and a noun. The former is live, doing, action and results oriented while as the latter seems to be about the position-passive. Kindness leadership needed now more than ever, in global health and beyond?
